# Optimizing Dietary Protein and Lipid Levels for Greenfin Filefish (*Thamnaconus septentrionalis*) in Practical Feeds: Effects on Growth Performance, Feed Utilization, and Physiological Status

**DOI:** 10.1155/anu/2928232

**Published:** 2026-10-22

**Authors:** Zhidong Song, Huadong Li, Peiyu Li, Lei Yu, Xihan Xu, Jian Zhang, Yuming Sun, Chengqiang Wang, Yige Wu, Xiaoyan Wang, Yongzhi Sun

**Affiliations:** ^1^ Shandong Marine Resource and Environment Research Institute Yantai 264006 China; ^2^ Shandong Shengsuo Feed Technology Co., Ltd. Yantai 265500 China; ^3^ Daoyi Human Resources Service Co., Ltd. Yantai 264003 China; ^4^ Shanghai Ocean University Shanghai 201306 China shou.edu.cn

**Keywords:** antioxidant capacity, growth performance, lipid requirement, oxidative stress, practical feed, protein requirement, *Thamnaconus septentrionalis*

## Abstract

The study investigated the optimal dietary protein and lipid levels for greenfin filefish (*Thamnaconus septentrionalis*) using two sequential, growth trial‐based experiments with practical formulations. Experiment I was designed to establish the quantitative protein requirement. Six isolipidic and isoenergetic diets with graded crude protein levels (38%, 41%, 44%, 47%, 50%, and 53% of dry matter) and a fixed lipid content of 10% were fed to triplicate groups of fish (5.56 g) for 56 days. Growth performance, feed utilization, and physiological responses were evaluated. Weight gain, specific growth rate, and feed conversion ratio all showed significant quadratic responses to dietary protein level, peaking at 47% protein. Quadratic regression analysis based on growth pinpointed the optimal dietary protein requirement at 45.93%–45.99%. This optimal level not only yielded the highest final biomass and best feed efficiency but was also associated with the highest hepatic total antioxidant capacity (T‐AOC), indicating an enhanced systemic antioxidant defense without provoking oxidative damage, as malondialdehyde (MDA) levels remained stable across all protein groups. Subsequently, Experiment II was conducted to determine the lipid requirement at the established optimal protein level (46%) by testing six isonitrogenous diets with graded lipid levels (6%, 7.5%, 9%, 10.5%, 12%, and 13.5%). Quadratic regression based on growth revealed an optimal dietary lipid range of 9.69%–10.39%. While lipid levels up to 10.5% promoted growth and improved feed conversion through a protein‐sparing effect, inclusion levels exceeding 12% induced metabolic dysfunction. This was evidenced by a sharp decline in T‐AOC and superoxide dismutase activity, a significant increase in MDA, elevated hepatosomatic index (HSI), and reduced lipid retention efficiency. Notably, the concurrent accumulation of hepatic unsaturated fatty acids and rise in MDA at high lipid intakes suggested these endogenous pools became substrates for peroxidation, overwhelming the antioxidant system. Therefore, a practical formulation containing 46% protein, 9.7%–10.4% lipid, and 33% carbohydrate, with a crude protein‐to‐gross energy ratio (CP/GE) of ~23.93–24.59 mg/kJ, is recommended for juvenile greenfin filefish (<20 g).

## 1. Introduction

The greenfin filefish *Thamnaconus septentrionalis*, an economically important species native to the Northwest Pacific, has seen a remarkable transition in China from a minor bycatch to a major aquaculture product. This shift was primarily driven by a severe decline in wild stocks, with annual catch plummeting from 20.97 tons in 2009 to merely 11.89 tons by 2024 [1]. In response, China has prioritized the development of artificial propagation and captive breeding technologies, leading to significant breakthroughs in larval rearing. These advances have enabled the rapid expansion of its aquaculture, establishing a sustainable alternative to wild fisheries. The species is predominantly cultured in marine net pens across coastal provinces such as Shandong, Fujian, and Zhejiang, where juveniles are stocked in spring and typically reach market size (300–500 g) within 8–12 months. Successful cultivation requires integrated management of multiple factors, including water quality, formulated feeding regimens, stocking density, and disease prevention, due to the species’ high sensitivity to environmental and husbandry conditions [2–4]. Despite its growing economic significance, the sustainable expansion of *T. septentrionalis* aquaculture faces a critical constraint: the absence of optimized, cost‐effective formulated feeds tailored to its specific nutritional needs. The industry has historically relied on frozen krill, which is ecologically unsustainable, expensive, and threatens wild krill populations. The development of practical formulated feeds has been hindered by a fundamental lack of species‐specific nutritional research. In particular, basic nutrient requirements—especially for protein and lipid, the two most critical macronutrients in aquafeeds—remain poorly defined.

As a promising species for marine aquaculture, the sustainable farming of filefish relies on the development of precision‐formulated feeds, which in turn depends on a robust understanding of species‐specific nutrient requirements. Protein, as the primary macronutrient for growth and metabolic functions, must be optimized within a narrow effective range [5]; deficiency impairs growth and health, while excess leads to inefficient nitrogen utilization and increased environmental pollution. Lipids, similarly, serve not only as a dense energy source but also as essential structural and functional components, and their imbalance can result in poor growth, health issues, or excessive lipid deposition [6]. Determining the precise requirements for both protein and lipid is thus critical to maximizing growth while minimizing aquaculture’s environmental impact [7]. Currently, the nutrient requirements for *T. septentrionalis* remain poorly defined. The study by Xu et al. [8] provided a valuable initial assessment by evaluating growth across nine protein–lipid combinations using a fishmeal–casein‐based diet. While this multilevel screening approach successfully identified a favorable nutrient ratio (43% protein and 7% lipid, CP/GE = 23.07 mg/kJ) under experimental conditions, it was not designed to establish the minimum quantitative requirement for either protein or lipid. Moreover, the use of refined ingredients such as casein does not reflect commercial aquafeeds, which rely on practical ingredients whose matrix effects, digestibility, and palatability can significantly alter nutrient utilization. Thus, the present study was conceived to address these critical informational gaps by systematically and concurrently investigating the optimal dietary protein and lipid levels for *T. septentrionalis* using practical feed formulations. The findings are expected to provide an initial nutritional framework that will support the nascent aquaculture industry for this species at an initial weight of ~5 g, promoting both economic viability and environmental stewardship.

## 2. Methods and Materials

### 2.1. Experimental Design

Two sequential feeding trials were conducted to determine the optimal dietary protein and lipid levels for juvenile greenfin filefish. The first trial (initiated on May 10, 2025) established the protein requirement using a practical diet. This optimal protein level then served as the basis for the second trial (initiated on July 12, 2025), which focused on lipid optimization. The overall experimental design is outlined in Figure 1.

**Figure 1:**
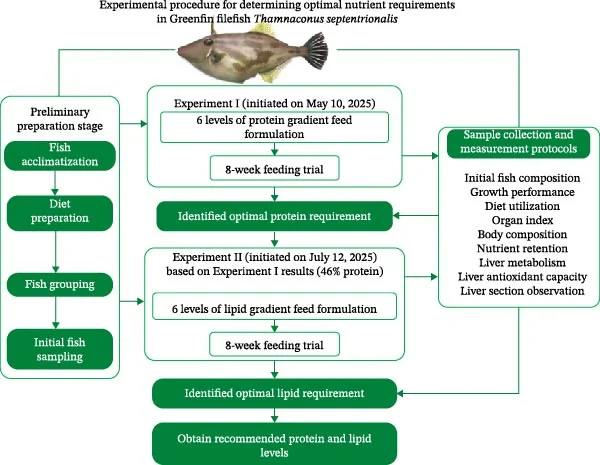
Flow chart of the two‐phase (Experiment I initiated on May 10, 2025 and Experiment II initiated on July 12, 2025) experimental design for protein and lipid optimization in greenfin filefish juveniles.

### 2.2. Diet Formulation and Preparation

#### 2.2.1. Experiment I Diets

Six isolipidic and isoenergetic diets were formulated to contain graded levels of crude protein: 38%, 41%, 44%, 47%, 50%, and 53% of dry matter (Table 1). This formulation contained ~50% fish meal as the primary protein source, with moderate inclusion of alternative proteins (soy protein concentrate, chicken meal, fermented peanut meal, soybean meal, and shrimp meal). Fish oil served as the sole lipid source across all diets. To isolate the effect of dietary protein, the lipid content was maintained constant at ~10% in all formulations. This was achieved by dynamically adjusting the inclusion level of fish oil and using microcrystalline cellulose as an inert filler to compensate for changes in the volume of protein ingredients. To enhance palatability, all diets were supplemented with fixed levels of sulfobetaine and taurine (0.8%). The formula, essential amino acid composition of the six diets with graded protein levels, is presented in Table 1‐and Figure 2a, which, despite variations in absolute content, reflects a constant amino acid pattern relative to the total protein.

**Figure 2:**
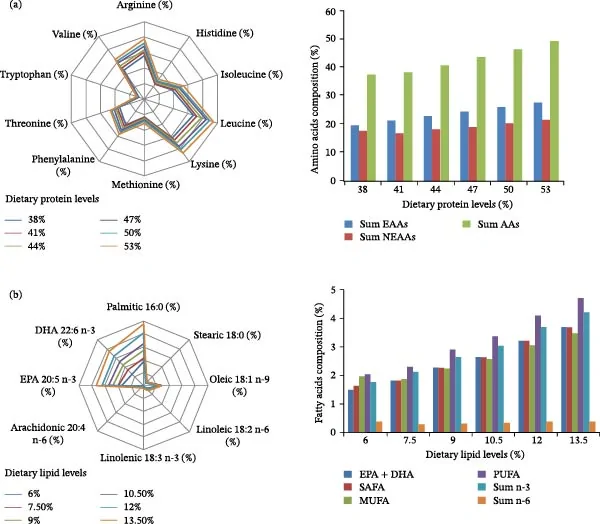
Essential amino acid and key fatty acid profiles of the experimental diets. (a) Essential amino acid composition of the six diets with graded protein levels. (b) Key fatty acid composition of the six diets with graded lipid levels. Both sets of diets exhibited similar patterns within their respective nutrient gradients. AA: Amino acid; DHA: Docosahexaenoic acid; EAA: Essential amino acid; EPA: Eicosapentaenoic acid; MUFA: Monounsaturated fatty acid; NEAA: Non‐essential amino acid; PUFA: Polyunsaturated fatty acid; SAFA: Saturated fatty acid.

**Table 1:** Formulation and ingredients of diets used in two experiments.

Ingredients (% dry basis)	Experiment Ⅰ						Experiment Ⅱ					
	Dietary protein levels (%)						Dietary lipid levels (%)					
	38	41	44	47	50	53	6	7.5	9	10.5	12	13.5
Fishmeal^a^	40.43	43.62	46.81	50.00	53.19	56.38	50.00	50.00	50.00	50.00	50.00	50.00
Soy protein concentrate^b^	9.70	10.47	11.23	12.00	12.77	13.53	12.00	12.00	12.00	12.00	12.00	12.00
Chicken meal^c^	2.43	2.62	2.81	3.00	3.19	3.38	3.00	3.00	3.00	3.00	3.00	3.00
Fermented peanut meal^d^	4.04	4.36	4.68	5.00	5.32	5.64	5.00	5.00	5.00	5.00	5.00	5.00
Soybean meal^e^	3.44	3.71	3.98	4.25	4.52	4.79	4.25	4.25	4.25	4.25	4.25	4.25
Shrimp meal^f^	2.43	2.62	2.81	3.00	3.19	3.38	3.00	3.00	3.00	3.00	3.00	3.00
Pregelatinized starch^g^	8.86	8.8	8.74	8.66	8.59	4.49	8.71	8.71	8.71	8.71	8.71	8.71
Fish oil^h^	5.93	5.56	5.2	4.84	4.48	4.12	0.34	1.84	3.34	4.84	6.34	7.84
Vitamin mineral premix^i^	1.00	1.00	1.00	1.00	1.00	1.00	1.00	1.00	1.00	1.00	1.00	1.00
Soy lecithin^j^	1.00	1.00	1.00	1.00	1.00	1.00	1.00	1.00	1.00	1.00	1.00	1.00
Sulfobetaine^k^	0.05	0.05	0.05	0.05	0.05	0.05	0.05	0.05	0.05	0.05	0.05	0.05
Dicalcium hydrogen phosphate^l^	0.2	0.2	0.2	0.2	0.2	0.2	0.2	0.2	0.2	0.2	0.2	0.2
Microcrystalline cellulose^m^	19.69	15.19	10.69	6.2	1.7	1.24	10.65	9.15	7.65	6.15	4.65	3.15
Taurine^n^	0.75	0.75	0.75	0.75	0.75	0.75	0.75	0.75	0.75	0.75	0.75	0.75
Ethoxyquin^o^	0.05	0.05	0.05	0.05	0.05	0.05	0.05	0.05	0.05	0.05	0.05	0.05
Total	100	100	100	100	100	100	100	100	100	100	100	100

Proximate composition (Measured value, % dry material)												
Crude protein	38.02	41.03	44.20	47.04	50.04	53.07	46.43	46.45	46.19	46.51	46.53	46.31
Crude lipid	10.08	10.14	10.26	10.35	10.73	10.19	6.11	7.65	8.99	10.35	11.93	13.38
Crude ash	8.18	9.34	10.26	10.08	11.70	10.92	11.09	11.51	11.60	10.08	10.72	10.07
Carbohydrate^p^	43.72	39.49	35.28	32.53	27.53	25.82	36.37	34.39	33.22	33.06	30.82	30.24
Gross energy (kJ/g)	19.45	19.27	19.14	19.19	18.77	18.96	18.13	18.38	18.65	19.03	19.31	19.74
CP/GE^q^ ratio (mg/kJ)	19.54	21.29	23.09	24.51	26.65	27.99	25.61	25.27	24.77	24.44	24.10	23.46
CP/CL^r^ ratio	3.77	4.05	4.31	4.54	4.66	5.21	7.60	6.07	5.14	4.50	3.90	3.46

^a^Crude protein, 64.5%; Crude lipid, 8.0%; Haida Marine Technology Co., Ltd., Dongying, China.

^b^Crude protein, 60.5%, Crude lipid, 3.0%; Dongwei Feed Co., Ltd., Rizhao, China.

^c^Crude protein 63.5%, Crude lipid, 12.0%; Hainuo Biotechnology Co., Ltd., Binzhou, China.

^d^Crude protein 51.0%, Crude lipid, 1.5%; Luhua Biological Protein Co., Ltd., Laiyang, China.

^e^Crude protein 43.5%, Crude lipid 1.5%; XiangchiFoodgrain&Cooking Oil Co., Ltd., Longkou, China.

^f^Crude protein 56.5%, Crude lipid 20.0%; Shanghai Runan Import & Export Co., Ltd., Shanghai, China.

^g^Purity 98%; Shuohui Food Co., Ltd., Tsingdao, China.

^h^Purity 98%; Shunhe Feed Co., Ltd., Rizhao, China.

^i^Purity 99.5%; Daynew Aquatic Sci‐Tech Co., Ltd., Guangdong, China.

^j^Acetone‐insoluble matter 98.3%; Meiyesway Biotechnology Co., Ltd., Hebei, China.

^k^Purity 98.0%; Yujin Trading Co., Ltd., Zibo, China.

^l^Purity 92.5%; New Vita Trading Co., Ltd., Jinan, China.

^m^Purity 99%; LinghuXinwang Chemical Co., Ltd., Huzhou, China.

^n^Purity > 95%; Yongan Pharmaceutical Co., Ltd., Qianjiang, China.

^o^Purity > 99%; Ruitai Chemical Co., Ltd., Taixing, China.

^p^Calculated on a dry basis as: 100 – (crude protein + crude lipid + crude ash).

^q^CP/CE ratio is crude protein/gross energy ratio (mg/kJ).

^r^CP/CL ratio is crude protein/crude lipid ratio.

#### 2.2.2. Experiment II Diets

Based on the optimal dietary protein range determined in Experiment I, six isonitrogenous practical diets were formulated to contain graded levels of crude lipid: 6%, 7.5%, 9%, 10.5%, 12%, and 13.5% of dry matter. The same blend of practical protein sources used in the Experiment I optimal diet was maintained. Fish oil was used as the primary lipid source. The varying lipid levels were achieved by adjusting the inclusion of fish oil, with microcrystalline cellulose used as a filler to maintain isoenergetic conditions and equalize the total mass of the diets. All other additives, including palatability enhancers, and the binder, were included at the same levels as in the Experiment I diets (Table 1). The fatty acid composition of the diets, which varied with the graded fat levels, is presented in Figure 2b and reflects a constant fatty acid profile relative to the total lipids.

#### 2.2.3. Diet Preparation

The preparation process was identical for both experiments. All dry ingredients were first ground into a fine powder, passed through an 80‐mesh sieve, and then thoroughly mixed in a twin‐cylinder mixer for 15 min. The fish oil was then added gradually and mixed for another 10 min to ensure homogeneity. Finally, ~30% (v/w) of deionized water was added to form a dough. The moist mixture was pelleted using a laboratory‐scale single‐screw extruder (Model TPE62, Jiangsu Yang Gong Machinery Technology Development Co., Ltd., Yangzhou, China) equipped with 1.3 and 2.0 mm dies. The extrusion temperature was maintained at ~95°C, and the screw speed was set to 215 rpm. The resulting pellets were steam‐dried in a convection oven at 45°C for 24 h until the moisture content was reduced to below 10%. After drying, the diets were sealed in airtight plastic bags and stored at −20°C until use to preserve nutrient integrity.

### 2.3. Feeding Trial Management

Although Experiment I (initiated on May 10, 2025) and Experiment II (initiated on July 12, 2025) were conducted sequentially, all management protocols were kept identical between the two trials to ensure data comparability, including water quality parameters, feeding regime, and stocking density.

#### 2.3.1. Fish Source and Acclimation

Healthy juvenile greenfin filefish with an initial body weight of ~5.47 g were obtained from a commercial hatchery (Yinze Biotechnology Co., Ltd., Weihai, China). During this period, they were fed a commercial marine carnivorous fish diet (Shandong Shengsuo Feed Technology Co., Ltd., China; crude protein: 52%, crude lipid: 12%; extruded pellet, pellet size: 1.0 mm) to satiation twice daily.

#### 2.3.2. Experimental System and Conditions

The trials were conducted in a recirculating aquaculture system, which consisted of 18 independent fiberglass tanks (300‐L capacity each). The environmental conditions were rigorously maintained throughout the experimental period as follows: salinity at 30 ± 1 ppt, water temperature at 24.0 ± 0.5°C, dissolved oxygen concentration above 6.0 mg/L, and a natural light–dark cycle (12 h light: 12 h dark). The flow rate of seawater to each tank was maintained at ~7.0 L/min. Aeration was supplied to each tank to maintain oxygen saturation. Water quality parameters (temperature, salinity, pH, and ammonia nitrogen) were monitored daily at 09:00 to ensure stability.

#### 2.3.3. Experimental Distribution and Feeding Regime

At the start of each trial (Day 0), fish were fasted for 24 h, anesthetized mildly with MS‐222 (3‐aminobenzoic acid ethyl ester methanesulfonate, 50 mg/L), individually weighed, and randomly distributed into the experimental tanks. Each dietary treatment was assigned to triplicate tanks (*n* = 3). For both experiments, the initial stocking density was ~1.3 kg/m^3^ (70 fish per tank).The fish were fed their respective experimental diets by hand to apparent satiation three times daily (at 07:30, 12:00, and 16:30). ~30 min after each feeding session, any uneaten feed was carefully siphoned out from each tank, collected on a fine mesh, oven‐dried at 105°C to constant weight, and weighed to calculate the actual feed intake (FI). Tanks were inspected twice daily (08:00 and 18:00) for any mortality. Dead fish were removed immediately, recorded, and weighed. The final biomass calculations for growth performance excluded the weight of deceased fish.

### 2.4. Sample Collection and Analytical Methods

#### 2.4.1. Sample Collection

After the 56‐day feeding period, all fish were fasted for 24 h to evacuate gut contents. Fish from each tank were collectively weighed to determine final biomass. Subsequently, 9 fish per tank (*n* = 27 per dietary treatment) were randomly selected, anesthetized with MS‐222 (100 mg/L), and entire viscera were carefully dissected and weighed to calculate viscerosomatic index (VSI). Liver, diegestive tract and dorsal white muscle tissues were excised. After weighing each sample individually (for organ indices analyses),aportion of each tissue was immediately flash‐frozen in liquid nitrogen and stored at −80°C for proximate composition (moisture, crude protein, crude lipid, and ash) and biochemical analyses (e.g., enzyme activities). For liver histology, sections (~3 mm × 3 mm × 2 mm) from the median lobe were fixed in 10 volumes of Davidson’s fixative for 24 h at 4°C. The fixed tissues were then transferred to 70% ethanol for long‐term storage until processing.

#### 2.4.2. Analytical Methods

The moisture, crude protein, crude lipid, and ash contents of the experimental diets and freeze‐dried muscle/liver tissues were determined following standard Association of Official Analytical Chemists [9] methods. Moisture was determined by oven‐drying at 105°C to constant weight. Crude protein (N × 6.25) was analyzed by the Kjeldahl method using an automated analyzer (Kjeltec 8400, FOSS, Sweden). Crude lipid was quantified by petroleum ether extraction using a Soxhlet apparatus. Ash content was determined by combustion in a muffle furnace at 550°C for 6 h.

Frozen liver tissues were homogenized in ice‐cold physiological saline. After centrifugation at 8000×*g* for 15 min at 4°C, the supernatant was collected, aliquoted, and kept on ice for immediate analysis or stored at −80°C for later use. The supernatant was used to measure key parameters including antioxidant enzyme activities (superoxide dismutase, SOD; glutathione peroxidase, GSH‐Px), total antioxidant capacity (T‐AOC), and the concentrations of malondialdehyde (MDA) and total protein. All these assays were performed using commercial colorimetric kits (Nanjing Jiancheng Bioengineering Institute, Nanjing, China) according to the manufacturer’s instructions. The concentration of polyunsaturated fatty acids (PUFA) was determined separately using a kit from Shanghai Zenke Biotechnology Co., Ltd. (Shanghai, China). In addition, the activities of several metabolic enzymes were analyzed. Fatty acid synthase (FAS) activity was measured with a kit from Shanghai Enzyme‐linked Biotechnology Co., Ltd. (Shanghai, China). The activities of acetyl‐CoA carboxylase (ACC), carnitine palmitoyltransferase 1 (CPT‐1), glutamate dehydrogenase (GDH), alanine aminotransferase (ALT), aspartate aminotransferase (AST), phosphoenolpyruvate carboxykinase (PEPCK), and phosphofructokinase‐1 (PFK‐1) were determined using commercial kits from Nanjing Jiancheng Bioengineering Institute.

Histological analysis: Fixed liver tissues were dehydrated through a graded ethanol series, embedded in paraffin, and sectioned at a thickness of 5 μm. The sections were then stained with hematoxylin and eosin (H&E) for morphological examination. To specifically assess hepatic steatosis (lipid accumulation), the stained sections were examined under a light microscope. Key histopathological features indicative of lipid deposition were evaluated, including the distribution of intracellular lipid vacuoles, hepatocellular ballooning, and the overall degree of lipid droplet accumulation within the cytoplasm.

### 2.5. Calculations and Statistical Analysis

#### 2.5.1. Growth Performance and Feed Utilization Calculations

The following key indices were calculated for each replicate tank (experimental unit) based on the recorded data:

Weight gain WG, g=Final mean body weight g−initial mean body weight g

Specific growth rate SGR,%/day=Ln Final mean body weight− Ln Initial mean body weight/Days of feeding×100,

Feed intake FI, g=Total consumed feed g/Fish number,

Daily feed intake DFI,%/d=100×Total dry matter intake/Experimental days×Initial body weight+Final body weight/2,

Feed conversion ratio FCR=Total dry feed intake g/Total wet weight gain g,

Survival rate SR,%=Final number of fish/Initial number of fish×100,

Hepatosomatic index HSI,%=Liver weight g/Final body weight g×100,

Digestive tract index DI,%=Digestive tract weight g/Final body weight g×100,

Viscerosomatic index VSI,%=Viscera weight g/Final body weight g×100,

Condition factors CF, g/cm3=Body weight/Body length3,

Protein retention efficiency (PRE, %)=Final weight×P12−Initial weight×PFeed intake×P,

Lipid retention efficiency (LRE, %)=Final weight×L12−Initial weight×LFeed intake×L,

Energy retention efficiency (ERE, %)=Final weight×E12−Initial weight×EFeed intake×E,

where *P*, *L*, and *E* represent the protein, lipid, and energy contents in the feed, while *P*1, *L*1, *E*1 and *P*2, *L*2, *E*2 denote these components in the final and initial fish bodies, respectively.

#### 2.5.2. Statistical Analysis

All data are presented as mean ± standard deviation (x ± s). The tank was considered the experimental unit for all analyses (*n* = 3 per dietary treatment). Statistical analyses were performed using SPSS Statistics software (Version 16.0, IBM Corp., USA).The effects of the dietary protein level and dietary lipid level on all response variables were analyzed separately using one‐way analysis of variance (ANOVA). Prior to ANOVA, all data were checked for assumptions of normality using the Shapiro–Wilk test and homogeneity of variances using Levene’s test. When a significant main effect was detected (*p* < 0.05), Duncan’s multiple range test was used for post hoc multiple comparisons among treatment means. To predict the optimal dietary protein or lipid requirement, the relationship between the nutrient level and the key growth performance parameter was fitted using a quadratic regression model. The significance level for all tests was set at*p* < 0.05.

## 3. Results

### 3.1. Growth Performance

In Experiment Ⅰ (Table 2), final weight, WG, and SGR were significantly influenced by dietary protein level (*p* < 0.05), with values increasing up to 47% protein and declining at higher levels. The highest final weight (18.61 g), WG (13.03 g), and SGR (2.27%/d) were observed at 47% dietary protein. SR did not differ across treatments (*p* > 0.05), whereas CF and FCR varied significantly (*p* < 0.05). The lowest FCR (0.85) was recorded at 47% protein. DFI was significantly lower in groups fed 44%–53% protein compared to those fed 38%–41% protein (*p* < 0.05). Quadratic regression analyses based on SGR and FCR indicated optimal dietary protein levels of 45.92% and 45.99%, respectively (Figure 3a,b). In addition, quadratic regression of SGR against dietary crude protein‐to‐gross energy (CP/GE) ratio yielded an optimal CP/GE of ~23.93 mg/kJ (Figure 3c), confirming that the 46% protein diet provides a balanced energy context for maximum growth.

**Figure 3:**
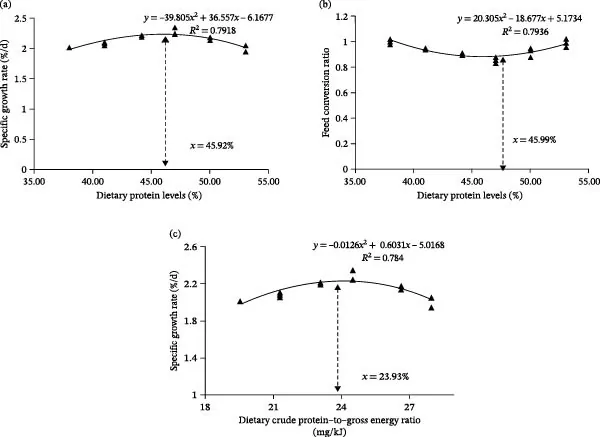
Optimal dietary protein level and optimal crude protein‐to‐gross energy (CP/GE) ratio determined by quadratic regression analysis. The panels show quadratic regressions of (a) specific growth rate (SGR), (b) feed conversion ratio (FCR) against graded dietary protein levels, and (c) SGR against dietary CP/GE ratio. The regression‐derived optimal dietary protein levels based on SGR and FCR were 45.93% and 45.99%, respectively, while the optimal P/E ratio for maximum growth was determined to be 23.98 mg/kJ.

**Table 2:** Growth performance of juvenile filefish fed the experimental diets (*n* = 3).

Experiment Ⅰ	Dietary protein levels (%)						*F*‐value	*p*‐Value
	38	41	44	47	50	53		
Growth								
Initial weight (g)	5.56 ± 0.01	5.56 ± 0.01	5.57 ± 0.03	5.57 ± 0.00	5.57 ± 0.00	5.57 ± 0.00	0.758	0.596
Final weight (g)	14.79 ± 0.14^a^	15.37 ± 0.27^ab^	17.90 ± 0.18^c^	18.61 ± 0.59^d^	17.43 ± 0.24^c^	15.95 ± 0.45^b^	57.511	0.000
WG (g)	10.56 ± 0.07^a^	11.14 ± 0.27^a^	12.33 ± 0.16^b^	13.03 ± 0.59^c^	11.86 ± 0.24^b^	10.60 ± 0.50^a^	23.353	0.000
SGR (%/d)	2.01 ± 0.01^a^	2.07 ± 0.03^b^	2.20 ± 0.01^b^	2.27 ± 0.06^c^	2.15 ± 0.03^b^	2.01 ± 0.06^a^	64.885	0.000
SR (%)	77.62 ± 6.75	83.33 ± 0.82	80.95 ± 1.65	82.38 ± 1.65	82.38 ± 4.36	85.24 ± 5.95	1.114	0.403
CF (g/cm^3^)	2.06 ± 0.17^b^	2.02 ± 0.21^b^	2.01 ± 0.25^b^	2.09 ± 0.36^b^	2.30 ± 0.25^a^	2.05 ± 0.21^b^	6.042	0.000
Diet utilization								
FCR	1.00 ± 0.01^c^	1.00 ± 0.02^c^	0.90 ± 0.01^ab^	0.85 ± 0.02^a^	0.91 ± 0.04^b^	0.93 ± 0.05^b^	12.703	0.000
FI (g/fish)	16.08 ± 0.29	15.73 ± 0.15	16.13 ± 0.27	15.88 ± 0.29	15.94 ± 0.83	15.92 ± 0.30	0.359	0.867
DFI (%/d)	2.60 ± 0.05^b^	2.59 ± 0.03^b^	2.41 ± 0.03^a^	2.31 ± 0.04^a^	2.43 ± 0.10^a^	2.44 ± 0.13^a^	6.863	0.003

**Experiment Ⅱ**	**Dietary lipid levels (%)**						***F*-value**	***p*-Value**
	**6**	**7.5**	**9**	**10.5**	**12**	**13.5**		

Growth								
Initial weight (g)	5.57 ± 0.00	5.57 ± 0.00	5.57 ± 0.00	5.57 ± 0.00	5.57 ± 0.00	5.57 ± 0.00	0.853	0.538
Final weight (g)	14.99 ± 0.44^a^	17.12 ± 0.68^c^	17.59 ± 0.39^cd^	18.31 ± 0.11^d^	16.07 ± 0.54^b^	15.18 ± 0.56^a^	22.781	0.000
WG (g)	9.42 ± 0.45^a^	11.55 ± 0.68^c^	12.02 ± 0.39^cd^	12.74 ± 0.11^d^	10.50 ± 0.54^b^	9.61 ± 0.56^a^	22.781	0.000
SGR (%/d)	1.87 ± 0.06^a^	2.12 ± 0.07^c^	2.17 ± 0.04^cd^	2.25 ± 0.01^d^	2.00 ± 0.06^b^	1.89 ± 0.07^a^	21.997	0.000
SR (%)	85.71 ± 2.47^b^	84.76 ± 4.59^b^	82.38 ± 1.65^b^	82.86 ± 2.47^b^	81.90 ± 2.18^b^	73.33 ± 0.82^a^	8.436	0.001
CF (g/cm^3^)	2.04 ± 0.19^b^	2.04 ± 0.22^b^	2.38 ± 0.20^a^	2.08 ± 0.26^b^	2.03 ± 0.19^b^	2.07 ± 0.21^b^	9.242	0.000
Diet utilization								
FCR	1.06 ± 0.06^e^	0.97 ± 0.02^d^	0.89 ± 0.01^b^	0.83 ± 0.00^a^	0.91 ± 0.03^bc^	0.96 ± 0.02^cd^	21.371	0.000
FI (g/fish)	15.88 ± 0.44^c^	15.65 ± 0.05^bc^	15.59 ± 0.33^bc^	15.25 ± 0.07^b^	14.68 ± 0.33^a^	14.57 ± 0.32^a^	10.168	0.001
DFI (%/d)	2.71 ± 0.13^d^	2.54 ± 0.04^c^	2.36 ± 0.03^b^	2.24 ± 0.01^a^	2.38 ± 0.06^b^	2.46 ± 0.02^bc^	20.200	0.000

*Note:* Values within a column with different superscript letters are significantly different (*p* < 0.05).

Abbreviations: CF, condition factor (g/cm^3^); DFI, daily feed intake (%/d); FCR, feed conversion ratio; FI, feed intake per fish (g); SGR, specific growth rate (%/d); SR, survival rate (%); WG, weight gain (g).

In Experiment Ⅱ, final weight, WG, and SGR were significantly affected by dietary lipid levels (*p* < 0.05), peaking at 10.5% lipid (WG: 12.74 g; SGR: 2.25%/d). SR differed significantly among treatments (*p* < 0.05), with the lowest SR (73.33%) observed at 13.5% dietary lipid level. CF also varied significantly (*p* < 0.05), reaching the highest value (2.38 g/cm³) at 9% lipid. FCR decreased with increasing lipid levels up to 10.5% (*p* < 0.05), achieving the minimum value (0.83). Both FI and DFI generally declined as dietary lipid levels increased (*p* < 0.05). Quadratic regression analyses of SGR and FCR against dietary lipid level yielded optimal points at 9.69% and 10.39% lipid, respectively (Figure 4a,b). Additionally, quadratic regression of SGR against dietary CP/GE ratio yielded an optimal CP/GE of ~24.59 mg protein/kJ (Figure 4c).

**Figure 4:**
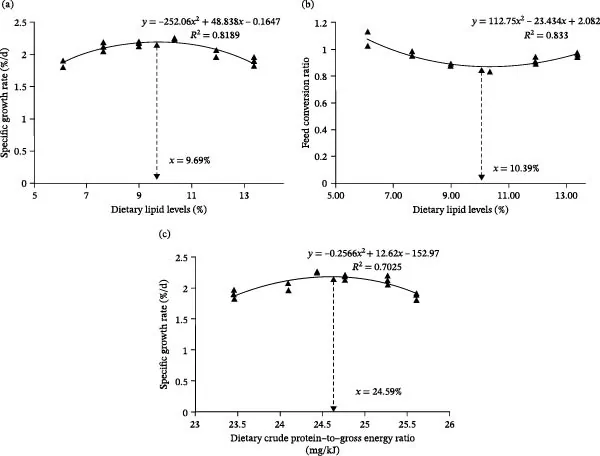
Optimal dietary lipid level and optimal crude protein‐to‐gross energy (CP/GE) ratio determined by quadratic regression analysis. The panels show quadratic regressions of (a) specific growth rate (SGR), (b) feed conversion ratio (FCR) against graded dietary lipid levels, and (c) SGR against dietary CP/GE ratio. The regression‐derived optimal dietary lipid levels based on SGR and FCR ranged from 9.69% to 10.39%, while the optimal CP/GE ratio for maximum growth was 24.59 mg/kJ.

### 3.2. Organosomatic Indices

In Experiment Ⅰ (Table 3), the DI showed a decreasing trend with increasing dietary protein levels, although the overall ANOVA did not detect a significant difference among all treatment means (*p* = 0.098). The HSI decreased significantly with increasing dietary protein levels (*p* < 0.05), with the highest value (11.10%) observed at 38% protein and the lowest (9.58%) at 53% protein. Similarly, the VSI also showed a significant declining trend (*p* < 0.05), with values decreasing from 17.07% at 38% protein to 15.82% at 53% protein.

**Table 3:** Organosomatic indices of juvenile filefish fed the experimental diets (*n* = 3).

Experiment Ⅰ	Dietary protein levels (%)						*F*‐value	*p*‐Value
	38	41	44	47	50	53		
DI (%)	5.19 ± 0.23	5.05 ± 0.33	4.98 ± 0.64	4.85 ± 1.12	4.84 ± 0.72	4.49 ± 0.83	1.909	0.098
HSI (%)	11.10 ± 0.94^c^	10.91 ± 1.12^c^	10.78 ± 1.43^bc^	10.64 ± 1.28^bc^	10.53 ± 1.12^ab^	9.58 ± 1.33^a^	4.101	0.002
VSI (%)	17.07 ± 1.09^c^	16.93 ± 1.41^c^	16.76 ± 1.47^bc^	16.53 ± 1.33^abc^	15.69 ± 1.69^a^	15.82 ± 1.47^ab^	2.763	0.021

**Experiment Ⅱ**	**Dietary lipid levels (%)**						***F*-value**	***p*-Value**
	**6**	**7.5**	**9**	**10.5**	**12**	**13.5**		

DI (%)	4.50 ± 0.57	4.59 ± 0.88	4.77 ± 0.94	5.04 ± 0.74	4.95 ± 0.57	5.02 ± 0.62	1.010	0.419
HSI (%)	8.59 ± 0.87^a^	9.23 ± 0.55^a^	10.51 ± 1.33^b^	10.60 ± 0.85^b^	10.90 ± 1.10^b^	10.85 ± 1.08^b^	9.390	0.000
VSI (%)	14.24 ± 0.92^a^	14.67 ± 1.01^a^	15.96 ± 1.08^b^	16.15 ± 1.21^b^	16.75 ± 0.78^b^	16.77 ± 0.83^b^	13.149	0.000

*Note:* Values within a column with different superscript letters are significantly different (*p* < 0.05).

Abbreviations: DI, digestive tract index (%); HSI, hepatosomatic index (%); VSI, viscerosomatic index (%).

In experiment Ⅱ, dietary lipid level did not significantly influence DI (*p* > 0.05). In contrast, both HSI and VSI increased significantly with increasing dietary lipid levels (*p* < 0.05). HSI increased from 8.59% at 6.0% lipid to 10.90% at 12.0% lipid, while VSI increased from 14.24% at 6.0% lipid to 16.77% at 13.5% lipid.

### 3.3. Nutrients Retention

In Experiment Ⅰ (Table 4), dietary protein level had a significant effect on protein utilization. Protein intake increased linearly with increasing dietary protein (*p* < 0.05). Both body protein accretion and PRE increased to a peak before declining as dietary protein levels rose, with the highest values at 44%–47% dietary protein. Quadratic regression analysis revealed that the PRE peaked at 44.18% dietary protein. Lipid intake did not differ among groups (*p* > 0.05), but body lipid accretion was significantly affected (*p* < 0.05), peaking at 47% protein. The LRE showed a increasing trend (*p* > 0.05), with the quadratic model indicating an optimum at 49.03% protein. Energy intake was not significantly different (*p* > 0.05), whereas both body energy accretion and ERE varied significantly (*p* < 0.05), with optimal values recorded at 44%–47% dietary protein and a regression‐based optimum at 47.72% protein (Figure 5).

**Figure 5:**
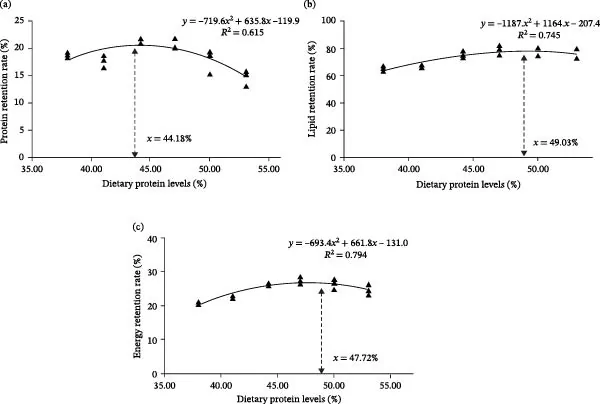
Optimal dietary protein level determined from nutrient retention efficiencies. (a) Quadratic regression of protein retention rate against dietary protein level, (b) quadratic regression of lipid retention rate against dietary protein level, (c) quadratic regression of energy retention rate against dietary protein level. The optimal levels were 44.18%, 49.03%, and 47.72%, respectively.

**Table 4:** Nutrient retention efficiency in filefish fed the experimental diets (*n* = 3).

Experiment Ⅰ	Dietary protein levels (%)						*F*‐value	*p*‐Value
	38	41	44	47	50	53		
Protein								
Protein intake (g)	6.11 ± 0.11^a^	6.45 ± 0.06^a^	7.13 ± 0.12^b^	7.47 ± 0.14^b^	7.97 ± 0.42^c^	8.45 ± 0.16^d^	57.783	0.000
Body protein accretion(g)	1.15 ± 0.02^a^	1.21 ± 0.01^a^	1.53 ± 0.04^b^	1.55 ± 0.04^b^	1.41 ± 0.13^b^	1.24 ± 0.10^a^	14.941	0.000
PRE (%)	18.80 ± 0.54^bc^	17.64 ± 1.13^b^	21.42 ± 0.55^d^	20.73 ± 0.97^cd^	17.78 ± 2.18^b^	14.71 ± 1.49^a^	10.809	0.000
Lipid								
Lipid intake (g)	1.62 ± 0.03	1.60 ± 0.02	1.66 ± 0.03	1.64 ± 0.03	1.61 ± 0.09	1.61 ± 0.11	0.875	0.526
Body lipid accretion (g)	1.05 ± 0.02^a^	1.07 ± 0.03^a^	1.26 ± 0.06^bc^	1.30 ± 0.04^c^	1.24 ± 0.03^bc^	1.21 ± 0.06^b^	17.935	0.000
LRE (%)	65.06 ± 1.13^a^	66.90 ± 1.31^a^	75.84 ± 2.56^b^	79.31 ± 3.50^b^	76.96 ± 3.37^b^	75.64 ± 4.03^b^	12.041	0.053
Energy								
Energy intake (kJ)	276.01 ± 4.96	268.10 ± 2.59	272.74 ± 4.59	270.15 ± 4.90	263.23 ± 13.72	268.77 ± 5.02	1.174	0.377
Body energy accretion (kJ)	59.00 ± 0.64^a^	60.41 ± 0.89^a^	72.85 ± 2.30^cd^	74.97 ± 2.02^d^	70.39 ± 1.32^c^	66.53 ± 3.50^b^	31.720	0.000
ERE (%)	21.38 ± 0.21^a^	22.53 ± 0.23^a^	26.71 ± 0.41^c^	27.76 ± 1.17^c^	26.79 ± 1.52^c^	24.76 ± 1.58^b^	18.430	0.000

**Experiment Ⅱ**	**Dietary lipid levels (%)**						***F*-value**	***p*-Value**
	**6**	**7.5**	**9**	**10.5**	**12**	**13.5**		

Protein								
Protein intake (g)	7.69 ± 0.21^c^	7.58 ± 0.03^bc^	7.51 ± 0.16^bc^	7.34 ± 0.03^ab^	7.13 ± 0.16^a^	7.13 ± 0.15^a^	8.464	0.001
Body protein accretion (g)	1.20 ± 0.09^b^	1.47 ± 0.17^c^	1.39 ± 0.08^c^	1.51 ± 0.03^c^	1.23 ± 0.08^b^	1.06 ± 0.05^a^	16.678	0.000
PRE (%)	15.65 ± 1.70^a^	19.36 ± 2.42^bc^	18.48 ± 0.88^bc^	20.65 ± 0.50^c^	17.25 ± 1.03^ab^	14.93 ± 0.84^a^	7.508	0.002
Lipid								
Lipid intake (g)	0.97 ± 0.03^a^	1.20 ± 0.00^b^	1.40 ± 0.03^c^	1.58 ± 0.01^d^	1.75 ± 0.04^e^	1.95 ± 0.04^f^	471.451	0.000
Body lipidaccretion (g)	0.78 ± 0.08^a^	1.06 ± 0.05^bc^	1.17 ± 0.08^d^	1.28 ± 0.06^e^	1.23 ± 0.03^cd^	1.03 ± 0.11^b^	49.629	0.000
LRE (%)	80.58 ± 11.29^c^	88.49 ± 3.78^c^	85.03 ± 5.03^c^	81.00 ± 4.01^c^	64.28 ± 1.83^b^	52.80 ± 5.16^a^	15.584	0.000
Energy								
Energy intake(kJ)	256.15 ± 7.03	256.36 ± 0.90	259.58 ± 5.45	262.11 ± 1.19	255.73 ± 5.72	259.77 ± 5.63	0.823	0.557
Body energy accretion (kJ)	49.48 ± 5.26^a^	64.43 ± 2.77^c^	67.12 ± 4.16^cd^	73.33 ± 2.63^d^	62.98 ± 1.11^c^	56.61 ± 3.93^b^	16.357	0.000
ERE (%)	19.35 ± 2.44^a^	25.13 ± 1.10^c^	25.85 ± 14.23^cd^	27.98 ± 1.03^d^	24.63 ± 0.30^c^	21.78 ± 1.09^b^	14.790	0.000

*Note:* Values within a column with different superscript letters are significantly different (*p* < 0.05).

Abbreviations: ERE, energy retention efficiency (%); LRE, lipid retention efficiency (%); PRE, protein retention efficiency (%).

In Experiment Ⅱ, protein intake decreased significantly with increasing dietary lipid levels (*p* < 0.05). Both body protein accretion and PRE increased to a peak before declining as dietary lipid level rose, with the highest values at 10.5% dietary lipid. Regression analysis indicated the optimal dietary lipid level for protein retention was 9.52%. As expected, lipid intake increased markedly with dietary lipid levels increasing (*p* < 0.05). Body lipid accretion increased up to 10.5% lipid and then declined (*p* < 0.05). LRE decreased significantly at higher lipid levels (12%–13.5%) compared to lower levels (6%–10.5%) (*p* < 0.05), with the quadratic optimum calculated at 8.05% lipid. Energy intake did not differ among groups (*p* > 0.05), but body energy accretion and ERE were significantly influenced (*p* < 0.05), with the highest values recorded at 9%–10.5% dietary lipid and a regression optimum at 10.02% lipid (Figure 6).

**Figure 6:**
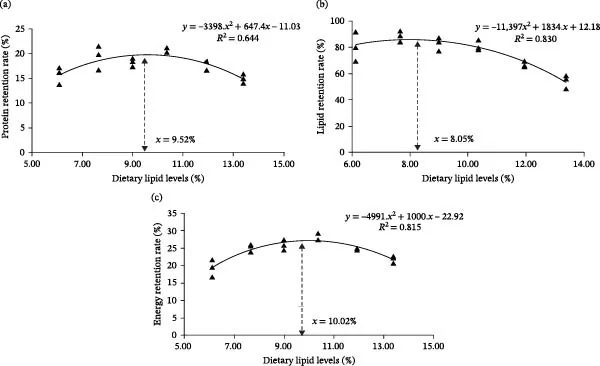
Optimal dietary lipid level determined from nutrient retention efficiencies. (a) Quadratic regression of protein retention rate (PRR) against dietary lipid level, (b) quadratic regression of lipid retention rate (LRR) against dietary lipid level, (c) quadratic regression of energy retention rate (ERR) against dietary lipid level. The optimal levels were 9.52%, 8.05%, and 10.02%, respectively.

### 3.4. Body Composition

In Experiment Ⅰ (Table 5), moisture and crude lipid contents of whole fish were not significantly affected by dietary protein levels (*p* > 0.05). Crude protein content increased significantly (*p* < 0.05), with the higher values observed at 44%–53% protein. Crude ash content also varied significantly (*p* < 0.05). In muscle, moisture and ash were unaffected, while crude protein content increased significantly with increasing dietary protein (*p* < 0.05). Conversely, muscle crude lipid content decreased significantly at higher protein levels, with the lowest value at 53% protein. In the liver, moisture content was significantly higher at 50% and 53% protein (*p* < 0.05), and crude lipid content showed a significant decreasing trend with increasing dietary protein (*p* < 0.05).

**Table 5:** Proximate composition of filefish fed experimental diets (*n* = 3).

Experiment Ⅰ	Dietary protein levels (%)						*F*‐value	*p*‐Value
	38	41	44	47	50	53		
Whole fish (% dry material)								
Moisture (% wet weight)	75.37 ± 0.46	75.55 ± 0.40	75.54 ± 0.32	75.53 ± 0.22	75.31 ± 0.33	75.41 ± 0.42	0.227	0.944
Crude protein	52.46 ± 0.56^a^	53.47 ± 2.26^ab^	55.81 ± 0.99^c^	54.66 ± 0.66^bc^	55.79 ± 2.47^c^	55.43 ± 1.56^bc^	4.424	0.004
Crude lipid	39.69 ± 3.14	38.77 ± 0.77	38.68 ± 1.36	38.33 ± 1.22	37.97 ± 0.71	38.24 ± 1.72	1.110	0.369
Crude ash	8.88 ± 0.05^a^	9.25 ± 0.18^ab^	10.13 ± 0.52^c^	9.82 ± 0.36^bc^	9.34 ± 0.91^ab^	9.79 ± 0.82^bc^	4.133	0.006
Muscle (% dry material)								
Moisuture (% wet weight)	76.32 ± 0.50	76.05 ± 0.20	76.86 ± 0.19	75.96 ± 0.80	76.15 ± 0.81	76.23 ± 0.65	0.892	0.516
Crude protein	75.39 ± 0.63^a^	76.74 ± 0.39^b^	77.13 ± 0.86^b^	77.55 ± 0.81^bc^	78.77 ± 1.07^d^	78.41 ± 0.87^cd^	13.299	0.000
Crude lipid	7.41 ± 0.18^bc^	7.57 ± 0.36^c^	7.47 ± 0.21^bc^	7.01 ± 0.04^b^	7.00 ± 0.32^b^	6.43 ± 0.33^a^	7.768	0.002
Crude ash	6.46 ± 0.09	6.42 ± 0.14	6.32 ± 0.03	6.66 ± 0.29	6.42 ± 0.27	6.68 ± 0.16	1.802	0.187
Liver (% dry material)								
Moisuture (% wet weight)	18.40 ± 0.80^a^	19.56 ± 0.67^ab^	20.05 ± 1.09^b^	18.72 ± 1.06^ab^	21.67 ± 0.62^c^	21.58 ± 0.74^c^	8.085	0.002
Crude protein	3.10 ± 0.22^a^	3.16 ± 0.14^ab^	3.33 ± 0.29^ab^	3.41 ± 0.11^ab^	3.49 ± 0.20^ab^	3.53 ± 0.21^b^	2.183	0.124
Crude lipid	94.71 ± 0.22^c^	94.68 ± 0.21^c^	94.47 ± 0.10^bc^	94.10 ± 0.33^ab^	94.10 ± 0.25^ab^	93.64 ± 0.41^a^	4.726	0.013
Crude ash	1.81 ± 0.05^a^	1.95 ± 0.05^b^	1.95 ± 0.08^b^	1.95 ± 0.06^b^	1.92 ± 0.06^ab^	1.96 ± 0.09^b^	2.035	0.145

**Experiment Ⅱ**	**Dietary lipid levels (%)**						***F*-value**	***p*-Value**
	**6**	**7.5**	**9**	**10.5**	**12**	**13.5**		

Whole fish (% dry material)								
Moisture (% wet weight)	76.53 ± 0.32^d^	75.99 ± 0.16^c^	75.86 ± 0.22^c^	75.60 ± 0.17^bc^	75.20 ± 0.40^ab^	74.86 ± 0.18^a^	15.881	0.000
Crude protein	57.91 ± 1.27^c^	57.41 ± 2.58^c^	54.00 ± 1.46^b^	54.72 ± 0.72^b^	54.78 ± 0.95^b^	50.96 ± 1.58^a^	14.201	0.000
Crude lipid	32.38 ± 1.80^a^	35.50 ± 1.99^b^	37.11 ± 1.60^bc^	38.33 ± 1.22^cd^	39.58 ± 1.58^d^	39.13 ± 2.24^d^	21.105	0.000
Crude ash	10.50 ± 0.57	9.95 ± 0.79	10.16 ± 0.61	9.82 ± 0.36	9.44 ± 1.07	9.59 ± 1.08	1.412	0.248
Muscle (% dry material)								
Moisuture (% wet weight)	76.26 ± 0.53	75.60 ± 0.57	76.33 ± 0.85	76.67 ± 0.49	76.20 ± 0.74	76.66 ± 0.26	1.266	0.340
Crude protein	79.98 ± 0.59^c^	80.82 ± 0.83^c^	79.53 ± 1.45^bc^	79.65 ± 1.03^bc^	78.46 ± 0.92^ab^	77.99 ± 0.13^a^	5.028	0.005
Crude lipid	7.31 ± 0.09^a^	8.18 ± 0.33^b^	8.38 ± 0.53^b^	8.61 ± 0.17^bc^	9.16 ± 0.45^c^	9.21 ± 0.41^d^	13.152	0.000
Crude ash	6.29 ± 0.04	6.41 ± 0.19	6.49 ± 0.11	6.29 ± 0.18	6.48 ± 0.04	6.41 ± 0.04	1.610	0.231
Liver (% dry material)								
Moisuture (% wet weight)	23.16 ± 0.94^b^	23.15 ± 0.87^b^	20.03 ± 0.69^a^	18.78 ± 1.37^a^	19.53 ± 1.23^a^	19.30 ± 0.99^a^	10.826	0.000
Crude protein	3.58 ± 0.15^c^	3.39 ± 0.18^bc^	3.09 ± 0.13^a^	2.99 ± 0.10^a^	3.07 ± 0.14^a^	3.19 ± 0.16^ab^	7.182	0.003
Crude lipid	94.07 ± 0.07^a^	94.23 ± 0.36^a^	94.61 ± 0.10^b^	94.76 ± 0.05^bc^	94.92 ± 0.33^bc^	95.07 ± 0.14^c^	10.032	0.001
Crude ash	1.86 ± 0.13	1.78 ± 0.11	1.87 ± 0.06	1.86 ± 0.10	1.92 ± 0.12	1.88 ± 0.04	0.636	0.677

*Note:* Values within a column with different superscript letters are significantly different (*p* < 0.05).

In Experiment Ⅱ, moisture content of whole fish decreased significantly (*p* < 0.05) while crude lipid content increased markedly (*p* < 0.05) with increasing dietary lipid levels. Crude protein content was significantly lower at higher lipid levels (9%–13.5%) (*p* < 0.05), and crude ash was not significantly affected (*p* > 0.05). In muscle, crude lipid content increased progressively with dietary lipid levels (*p* < 0.05), whereas crude protein content showed a significant decline at the highest lipid levels (*p* < 0.05). Moisture and ash were not significantly different (*p* > 0.05). In the liver, crude lipid content increased significantly (*p* < 0.05), and crude protein content was lower in groups fed 9%–12% lipid compared to the 6% lipid group (*p* < 0.05). Moisture content was also significantly affected (*p* < 0.05).

### 3.5. Liver Metabolic Enzymes Activities

Regarding lipid metabolism in Experiment Ⅰ (Table 6), neither FAS nor ACC activity was significantly affected by dietary protein levels (*p* > 0.05). However, CPT‐1 activity increased significantly with increasing protein levels (*p* < 0.05), reaching the highest values at 47%–50% protein. For protein metabolism, GDH and ALT activities were significantly elevated at protein levels of 44% and above (*p* < 0.05), whereas AST activity did not differ among groups (*p* > 0.05). In glucose metabolism, PEPCK activity increased progressively with dietary protein levels (*p* < 0.05), while PFK‐1 activity was significantly higher at 50% and 53% protein compared to lower protein groups (*p* < 0.05).

**Table 6:** Hepatic metabolic enzymes activities in filefish fed the experimental diets (*n* = 3).

Experiment Ⅰ	Dietary protein levels (%)						*F*‐value	*p*‐Value
	38	41	44	47	50	53		
Lipid metabolism								
FAS (nmol/min/mg prot)	17.89 ± 1.07	19.22 ± 1.69	20.10 ± 0.92	20.32 ± 1.72	18.76 ± 0.56	19.14 ± 1.27	1.466	0.271
ACC (U/mg prot)	3.39 ± 0.22	3.46 ± 0.15	3.60 ± 0.13	3.53 ± 0.07	3.55 ± 0.11	3.58 ± 0.04	1.132	0.395
CPT‐1 (nmol/min/mg prot)	13.84 ± 0.76^a^	14.69 ± 1.02^ab^	15.33 ± 0.64^bc^	15.97 ± 0.21^c^	15.98 ± 0.30^c^	15.50 ± 0.49^bc^	5.148	0.009
Protein metabolism								
GDH (nmol/min/mg prot)	355.62 ± 10.70^a^	359.88 ± 1.45^ab^	378.56 ± 2.13^c^	377.53 ± 9.87^c^	380.40 ± 6.13^c^	371.08 ± 1.28^bc^	7.640	0.002
ALT (U/mg prot)	211.50 ± 9.04^a^	215.80 ± 8.41^a^	235.99 ± 9.23^b^	237.05 ± 8.56^b^	234.17 ± 5.25^b^	232.93 ± 3.11^b^	5.057	0.010
AST (U/mg prot)	162.70 ± 11.97	171.57 ± 2.41	167.87 ± 2.92	175.31 ± 6.28	168.61 ± 9.03	175.99 ± 7.06	1.378	0.299
Glucose metabolism								
PEPCK (nmol/min/mg prot)	7.32 ± 0.37^a^	8.31 ± 0.36^b^	8.55 ± 0.20^bc^	8.82 ± 0.31^bc^	8.84 ± 0.43^bc^	9.10 ± 0.11^c^	11.899	0.000
PFK‐1 (U/mg prot)	82.20 ± 1.22^ab^	80.32 ± 1.99^a^	79.78 ± 3.82^a^	82.23 ± 0.99^ab^	87.37 ± 2.14^c^	86.01 ± 2.06^bc^	5.673	0.007

**Experiment Ⅱ**	**Dietary lipid levels (%)**						***F*-value**	***p*-Value**
	**6**	**7.5**	**9**	**10.5**	**12**	**13.5**		

Lipid metabolism								
FAS (nmol/min/mg prot)	22.07 ± 0.98^c^	21.11 ± 0.26^bc^	20.68 ± 0.63^abc^	20.62 ± 1.72^abc^	19.63 ± 0.64^ab^	19.34 ± 0.68^a^	3.624	0.031
ACC (U/mg prot)	3.86 ± 0.27^c^	3.65 ± 0.12^bc^	3.58 ± 0.07^ab^	3.48 ± 0.05^ab^	3.38 ± 0.04^a^	3.37 ± 0.10^a^	6.054	0.005
CPT‐1 (nmol/min/mg prot)	14.97 ± 0.19^a^	15.12 ± 0.23^a^	15.04 ± 0.36^a^	15.50 ± 0.49^ab^	15.74 ± 0.45^ab^	16.56 ± 1.16^b^	3.281	0.043
Protein metabolism								
GDH (nmol/min/mg prot)	386.09 ± 4.39^a^	376.58 ± 4.64^b^	370.60 ± 5.71^ab^	374.02 ± 5.97^ab^	367.95 ± 6.47^ab^	366.29 ± 3.74^a^	5.668	0.007
ALT (U/mg prot)	248.55 ± 10.45^b^	243.68 ± 7.12^b^	240.34 ± 4.92^b^	221.21 ± 6.57^a^	224.46 ± 5.16^a^	222.38 ± 3.83^a^	9.822	0.001
AST (U/mg prot)	183.05 ± 8.46^c^	179.76 ± 4.86^bc^	170.99 ± 3.77^ab^	169.41 ± 5.66^ab^	161.20 ± 6.99^ab^	162.55 ± 5.43^a^	6.418	0.004
Glucose metabolism								
PEPCK (nmol/min/mg prot)	8.74 ± 0.21	8.98 ± 0.29	8.74 ± 0.30	8.82 ± 0.31	8.92 ± 0.24	8.62 ± 0.17	0.777	0.585
PFK‐1 (U/mg prot)	73.57 ± 2.87^b^	74.57 ± 2.08^b^	71.24 ± 3.88^ab^	66.55 ± 2.88^a^	69.55 ± 1.37^ab^	66.32 ± 2.20^a^	5.081	0.010

*Note:* Values within a column with different superscript letters are significantly different (*p* < 0.05).

Abbreviations: ACC, acetyl‐CoA carboxylase (U/mg prot); ALT, alanine aminotransferase (U/mg prot); AST, aspartate aminotransferase (U/mg prot); CPT‐1, carnitine palmitoyltransferase‐I (nmol/min/mg prot); FAS, fatty acid synthetase (nmol/min/mg prot); GDH, glutamate dehydrogenase (nmol/min/mg prot); PEPCK, phosphoenolpyruvate carboxykinase (nmol/min/mg prot); PFK‐1, phosphofructokinase‐1 (U/mg prot).

In Experiment Ⅱ, both FAS and ACC activities decreased significantly with increasing dietary lipid levels (*p* < 0.05). In contrast, CPT‐1 activity showed an increasing trend, with the highest value observed at 13.5% lipid (*p* < 0.05). In protein metabolism, the activities of GDH, ALT, and AST all decreased significantly as dietary lipid levels increased (*p* < 0.05). For glucose metabolism, PEPCK activity was not significantly affected (*p* > 0.05), whereas PFK‐1 activity varied significantly (*p* < 0.05), with the highest activity observed at the lower lipid levels (6% and 7.5%) and a significant decrease at the higher lipid levels (10.5% and 13.5%).

### 3.6. Hepatocyte Antioxidant Capacity

In experiment Ⅰ (Table 7), hepatic T‐AOC was significantly affected by dietary protein levels (*p* < 0.05). The highest T‐AOC value was observed in fish fed a 47% protein diet, which was significantly higher than those fed the 38% and 41% protein diets. The activity of GSH‐Px also varied significantly among groups (*p* < 0.05), showing an increasing trend up to 44% protein, followed by a decrease at higher protein levels. In contrast, SOD activity, hepatic UFA content, and MDA concentration were not significantly influenced by dietary protein levels (*p* > 0.05).

**Table 7:** Hepatic antioxidant capacity in filefish fed the experimental diets (*n* = 3).

Experiment Ⅰ	Dietary protein levels (%)						*F*‐value	*p*‐Value
	38	41	44	47	50	53		
T‐AOC (U/mg prot)	0.33 ± 0.01^ab^	0.32 ± 0.02^a^	0.35 ± 0.01^bc^	0.36 ± 0.01^c^	0.34 ± 0.01^bc^	0.35 ± 0.01^bc^	6.579	0.004
SOD (U/mg prot)	227.23 ± 5.50	230.05 ± 4.82	235.04 ± 6.53	237.49 ± 7.41	235.58 ± 7.19	224.64 ± 8.10	1.612	0.231
GSH‐Px (U/mg prot)	397.64 ± 7.19^a^	427.16 ± 9.29^ab^	437.09 ± 4.75^b^	431.74 ± 5.81^b^	416.99 ± 6.49^a^	412.83 ± 11.46^a^	10.176	0.001
PUFA(μg/mg prot)	393.68 ± 7.54	384.02 ± 7.61	388.70 ± 5.97	390.88 ± 4.20	386.36 ± 4.36	373.98 ± 13.12	2.418	0.098
MDA (nmol/mg prot)	0.54 ± 0.06	0.58 ± 0.05	0.54 ± 0.06	0.51 ± 0.03	0.52 ± 0.06	0.49 ± 0.03	1.170	0.379

**Experiment Ⅱ**	**Dietary lipid levels (%)**						***F*-value**	***p*-Value**
	**6**	**7.5**	**9**	**10.5**	**12**	**13.5**		

T‐AOC (U/mg prot)	0.39 ± 0.01^c^	0.39 ± 0.00^bc^	0.38 ± 0.01^b^	0.36 ± 0.01^a^	0.35 ± 0.01^a^	0.35 ± 0.00^a^	32.881	0.000
SOD (U/mg prot)	231.57 ± 4.53^b^	234.52 ± 10.57^b^	237.38 ± 3.60^b^	237.65 ± 5.46^b^	227.51 ± 1.69^b^	213.55 ± 4.32^a^	7.544	0.002
GSH‐Px (U/mg prot)	428.91 ± 9.10^b^	419.00 ± 6.71^ab^	421.99 ± 7.60^ab^	415.54 ± 9.35^ab^	411.55 ± 5.77^a^	412.87 ± 3.10^a^	2.343	0.105
PUFA (μg/mg prot)	359.37 ± 4.37^a^	369.31 ± 3.95^a^	374.87 ± 9.27^bc^	377.16 ± 11.31^bc^	385.63 ± 5.81^c^	384.51 ± 3.76^bc^	5.889	0.006
MDA (nmol/mg prot)	0.43 ± 0.04^a^	0.51 ± 0.06^a^	0.53 ± 0.05^a^	0.53 ± 0.06^a^	0.69 ± 0.07^b^	0.73 ± 0.05^b^	14.136	0.000

*Note:* Values within a column with different superscript letters are significantly different (*p* < 0.05).

Abbreviations: GSH‐Px, glutathione peroxidase (U/mg prot); MDA, malondialdehyde (nmol/mg prot); PUFA, polyunsaturated fatty acids (μg/mg prot); SOD, superoxide dismutase (U/mg prot); T‐AOC, total antioxidant capacity (U/mg prot).

In Experiment II, the dietary lipid levels markedly influenced the hepatic antioxidant status and oxidative damage. T‐AOC decreased progressively with increasing dietary lipid from 6% to 13.5% (*p* < 0.05). SOD activity was significantly lower in the 13.5% lipid group compared to all other groups (*p* < 0.05). GSH‐Px activity was also significantly affected (*p* < 0.05), showing a decreasing trend at higher lipid levels. Concurrently, hepatic UFA content increased significantly with increasing dietary lipid level (*p* < 0.05), with the highest values observed in the 12% lipid group, which were significantly greater than those in the lower lipid groups (6% and 7.5%). Conversely, MDA content increased significantly in these two high‐lipid groups (12% and 13.5%) compared to groups fed 6% to 10.5% lipid (*p* < 0.05).

### 3.7. Hepatic Histology

Histological examination of liver sections revealed that dietary protein and lipid levels significantly influenced hepatic lipid accumulation (steatosis). As shown in Figure 7, increasing the dietary protein level from 38% to 53% led to a gradual reduction in the number and size of large lipid vacuoles within hepatocytes, accompanied by progressively darker tissue staining. This indicates a decrease in intracellular lipid deposition. Notably, at protein levels of 44% and above, lipid vacuoles became sparser and hepatocyte morphology appeared more compact, suggesting that high‐protein diets help reduce hepatic lipid storage.

**Figure 7:**
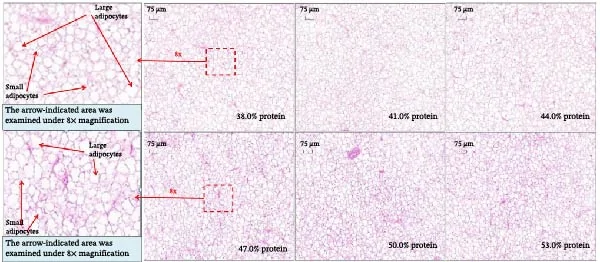
The effect of dietary protein level on the distribution of adipocytes in the liver. In the figure, as the dietary protein level increases from 38% to 41%, the adipocytes show no significant change. However, when the protein level rises to 44% or higher, large adipocytes tend to decrease, resulting in gradually deeper staining.

In contrast, Figure 8 demonstrates that elevating dietary lipid levels from 6% to 13.5% resulted in a marked increase in both the number and size of lipid vacuoles, with correspondingly lighter tissue staining. When the lipid level reached 12%, numerous large vacuoles were evident, and hepatocytes exhibited ballooning degeneration, indicating pronounced hepatic steatosis. These observations suggest that high‐lipid diets promote excessive intracellular lipid accumulation, potentially leading to metabolic burden and structural alterations in the liver.

**Figure 8:**
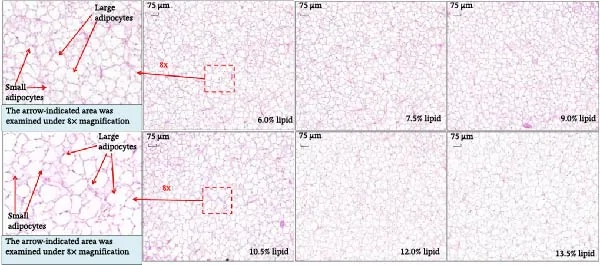
The effect of dietary fat level on the distribution of hepatic adipocytes. As shown in the figure, with an increase in dietary fat level, there is a tendency for large adipocytes to increase in number, resulting in progressively lighter staining. When the dietary fat level reaches >10.5%, large adipocytes become obviously more numerous and larger in size.

## 4. Discussion

### 4.1. Growth Performance

In Experiment I, quadratic regression analysis based on WG, SGR, and FCR yielded a mean optimal dietary protein level of ~46% for juvenile greenfin filefish, a prediction well (all *R* ^2^ > 0.6, which shows excellent fit) corroborated by the observed growth peak at 47% protein. This value is notably higher than the 43% reported by Xu et al. [8] for the same species and the 45% reported for the closely related *Stephanolepis cirrhifer* by Khosravi and Lee [10]. Given that the initial body weights of fish were comparable across studies (5.4 to 6.47 g), the discrepancy is most plausibly attributed to differences in dietary protein sources rather than ontogenetic shifts in requirements. Xu et al. [8] used a fish meal–casein combination, and Khosravi and Lee [10] employed a high fishmeal diet, both providing highly digestible protein with balanced amino acid profiles. In contrast, the practical protein blend used here contained substantial proportions of plant proteins and chicken meal, which generally exhibit lower digestibility and less balanced amino acid profiles as well as poor palatability, as reported in most marine species, where these protein sources were shown to be substantially less digestible than fish meal and casein [11–13]. Additionally, the 47% protein group, which exhibited the best feed efficiency, did not show a significant increase in total FI compared to other groups; its DFI was significantly lower than that of the low‐protein groups (38%–41%). This suggests that near the optimal protein level, nutrients were more efficiently utilized for growth rather than growth simply being driven by increased FI. The decline in both growth performance and FCR at protein levels above 47% indicates the onset of inefficient protein metabolism, where excess amino acids are likely deaminated for energy, increasing metabolic load and nitrogen excretion.

In Experiment II, dietary lipid levels exerted a significant dose‐dependent influence on growth, feed utilization, and survival. SGR and FCR followed a classic parabolic response, peaking at 10.5% lipid, a pattern confirmed by quadratic regression analyses yielding optimal points of 9.69% and 10.39% for SGR and FCR, respectively. The close alignment of these predicted optima (~9.69%–10.39%) with the observed peak at 10.5% strongly indicates that a dietary lipid level of ~10.0% is optimal under the present conditions. The improvement in growth and feed efficiency up to 10.5% lipid is primarily attributable to the protein‐sparing effect of lipids: as a dense, readily metabolizable energy source, adequate lipid reduces amino acid catabolism for energy, directing more ingested protein toward somatic growth [14]. This is reflected in the minimum FCR (0.83) at 10.5% lipid. Conversely, the decline in growth and the worsening of FCR at higher lipid levels (12% and 13.5%) signal nutritional imbalance and metabolic stress, as excessive lipid may overwhelm hepatic metabolism, leading to steatosis and disrupted nutrient homeostasis [15]. The progressive decline in FI and DFI with increasing lipid suggests an attempt to regulate energy intake [16], yet this regulation was insufficient to prevent adverse effects. Notably, the survival rate was lowest (73.33%) at 13.5% lipid; although the specific cause warrants further investigation, this level may have induced metabolic stress through oxidative damage or lipid overload, as supported by the significant decline in antioxidant capacity and elevated MDA levels observed at this dietary lipid level.

A key strength of this study is that Experiment II was conducted under the optimal protein level (46%) established in Experiment I, thereby isolating the lipid effect from confounding interactions with protein status [17]. Integrating both experiments, a dietary formulation containing ~46%–47% protein and 9.5%–10.5% lipid appears optimal for juvenile greenfin filefish, corresponding to a CP/GE ratio of 23.93–24.59 mg/kJ. Notably, this value is highly consistent with the CP/GE ratio of 23.07 mg/kJ reported for the same species by Xu et al. [8] and with 23.8 mg/kJ for the closely related *Stephanolepis cirrhifer* [10], reinforcing the reliability of our findings. However, this range is slightly lower than those reported for some omnivorous species, such as black sea bream *Sparus macrocephalus* (26.38 mg/kJ; [18]) and clownfish *Amphiprion sebae* (27–28 mg/kJ; [19]), suggesting that greenfin filefish may utilize nonprotein energy sources more efficiently, thus requiring a lower CP/GE ratio than these species.

### 4.2. Organosomatic Indices and Hepatic Histology

Analysis of organosomatic indices provides crucial physiological insights into how dietary protein and lipid levels differentially alter nutrient partitioning between growth, storage, and metabolic load in fish [20–22]. In Experiment I, as dietary protein increased from 38% to 53%, HSI and VSI decreased significantly, while DI showed a decreasing trend that did not reach statistical significance (*p* = 0.098). This marked reduction in relative liver and visceral mass with rising protein is consistent with observations in marine fish species fed high‐protein diets, such as Atlantic cod *Gadus morhua* ([23]), haddock *Melanogrammus aeglefinu* [24], and Atlantic halibut *Hippoglossus hippoglossus* [25]. The decline in organosomatic indices is closely linked to metabolic nutrient partitioning and is further supported by whole‐body and tissue composition data (Table 6). Muscle crude lipid decreased significantly and liver crude lipid showed a declining trend with increasing protein, whereas whole‐body and muscle crude protein increased. When dietary protein exceeds the requirement for somatic growth and maintenance, surplus amino acids are catabolized for energy via deamination and the urea cycle [26, 27]. The energy expended in this process may be partly derived from visceral and hepatic energy reserves, as reflected by lower tissue lipid levels and reduced organ mass, thereby contributing to the observed decreases in HSI and VSI.

In Experiment II, as dietary lipid rose from 6.5% to 14%, HSI increased from 8.59% to over 10.5% at ≥9.5% lipid, and VSI from 14.24% to above 16%, indicating that lipid exceeding metabolic demands was channeled into storage as triglycerides in the liver and visceral depots, as found in low lipid‐tolerant marine species such as haddock [28] and cobia *Rachycentron canadum* [29]. Two observations qualify this lipid deposition: lipid retention efficiency declined at 12%–13.5% lipid, and the rise in hepatic lipid was modest compared to the substantial increase in visceral and whole‐body lipid. This may suggest that hepatic storage capacity approached saturation, with surplus lipid increasingly diverted to the visceral cavity, as reflected by the progressive rise in VSI.

Histological examination confirmed these organometric changes. The increasing size and number of hepatic lipid vacuoles with rising dietary lipid levels correspond directly with elevated HSI values, whereas the reduction in lipid vacuoles at higher protein levels aligns with the declining HSI observed in Experiment I. This morphological correlation reinforces that changes in liver mass were primarily driven by alterations in lipid storage rather than functional tissue hypertrophy. The organosomatic and histological data demonstrate that increasing dietary protein reduced hepatic lipid storage, whereas increasing dietary lipid promoted progressive lipid accumulation in both liver and visceral depots, confirming that organ mass changes reflected alterations in lipid storage rather than tissue hypertrophy.

### 4.3. Body Composition

In Experiment I, elevating dietary protein from 38% to 53% induced a significant nutrient repartitioning effect, characterized by a concurrent rise in crude protein and a decline in lipid content in both whole body and muscle. This inverse relationship between protein and lipid deposition has been consistently documented in diverse species [17, 30–32], suggesting that higher protein intake was preferentially channeled into somatic protein synthesis, enhancing lean muscle mass. Under high‐protein conditions, lipids may be catabolized to fuel the metabolic cost of amino acid assimilation and ureagenesis [27], thus reducing the amount of lipid available for storage. Furthermore, the significant reduction in hepatic crude lipid directly correlates with and explains the declining HSI, implicating that high‐protein diets deplete hepatic endogenous lipid reserves to support the heightened energy demands of protein turnover.

At the established optimal protein level, the dietary lipid gradient revealed distinct effects on nutrient deposition (Experiment II). Whole‐body crude lipid increased linearly with rising dietary lipid, directly reflecting elevated lipid intake. Muscle lipid also showed a progressive, albeit gradual, increase, indicating that surplus lipid infiltrates the fillet beyond visceral storage. The most pronounced alteration occurred in the liver: hepatic crude lipid increased significantly, accompanied by decreased moisture and crude protein, signifying marked lipid accumulation and a metabolic shift toward lipid processing—consistent with observations in other species [28, 33, 34]. This biochemical profile validates the elevated HSI values. For filefish, a species traditionally valued for its liver oil [35], this may enhance natural storage capacity and potentially increase oil yield. However, the concomitant decrease in hepatic protein suggests a reprioritization of liver function, with resources diverted from protein synthesis toward lipid processing. A critical finding integrating across tissues is the significant decrease in whole‐body and muscle crude protein at the highest lipid levels (12% and 13.5%). This “nutrient dilution” effect [36] occurs when excessive lipid expands body lipid reserves, diluting the percentage of other constituents even if their absolute accretion is maintained. This shift toward a higher lipid‐to‐protein ratio underscores a fundamental drawback of high‐energy diets: they can maximize biomass gain while compromising the protein content and nutritional density of the harvestable product.

### 4.4. Nutrient Retention

The nutrient retention data from Experiment I reveal distinct patterns for protein, lipid, and energy utilization in response to graded dietary protein levels. As classically demonstrated in nutrient requirement studies [37], PRE exhibited a clear quadratic response, peaking at 44%–47% dietary protein with a calculated optimum of 44.18%‐the point of maximum efficiency in converting ingested protein into somatic protein. The significant decline in PRE beyond this optimum, despite a linear increase in protein intake, signals a metabolic shift where surplus amino acids are deaminated and catabolized for energy rather than incorporated into tissue [14, 27], directly explaining the growth plateau and subsequent decline at higher protein levels. In contrast, LRE demonstrated remarkable stability across 44%–53% protein, suggesting that lipid metabolism remained highly efficient and was not directly limited by dietary protein. This may involve priority‐based metabolic partitioning: when protein exceeds synthesis requirements (>47%), catabolized amino acids provide carbon skeletons that can support de novo lipogenesis, maintaining high lipid deposition as an alternative energy storage pathway [7, 38]. Thus, lipid deposition acts as a flexible “metabolic buffer” for excess energy and carbon. ERE integrated both trends, being highest at 44%–47% protein where PRE and LRE were synergistically maximized. Notably, ERE remained high at 50% protein: although PRE had begun to decline, persistently high LRE compensated for the energetic inefficiency of increased protein catabolism—a critical metabolic trade‐off [39]. However, at 53% protein, the substantial decline in PRE, coupled with a possible ceiling on compensatory lipid storage, resulted in a significant ERE drop.

In Experiment II, conducted at the fixed optimal protein level of 46%, lipid intake increased dramatically, but LRE significantly decreased at 12%–13.5% lipid. This decline in lipid storage efficiency, coupled with plateaued and then decreased body lipid accretion beyond 10.5% lipid, indicates saturation of lipid deposition pathways or upregulation of lipid oxidation; energy not efficiently stored likely contributes to increased metabolic heat increment and reduced growth at high lipid levels. Importantly, protein retention was significantly affected by dietary lipid, with the highest PRE at 10.5% lipid (regression optimum at 9.52%), confirming the protein‐sparing effect of dietary lipid [14]. Adequate dietary lipid provides readily available energy, reducing amino acid catabolism and sparing more for protein synthesis. However, at 12%–13.5% lipid, PRE declined, possibly due to metabolic overload disrupting amino acid uptake or protein synthesis pathways. ERE peaked at 9%–10.5% lipid (optimum 10.02%), mirroring growth and feed efficiency trends, indicating that dietary energy from the balanced protein–lipid combination was most efficiently captured as body mass within this range. Collectively, the retention data reveal that maximal growth occurred at a lipid level (9.5%–10.5%) slightly above that for maximal lipid storage efficiency (8.1%), indicating a metabolic trade‐off where moderately higher lipid supports protein sparing and growth despite marginally reduced lipid retention.

### 4.5. Hepatic Metabolic Enzymes

Analysis of key hepatic enzyme activities provides a molecular rationale for the patterns of nutrient utilization, growth, and body composition observed in the preceding experiments. In Experiment I, the primary metabolic adjustment to increasing dietary protein was a significant upregulation of amino acid catabolic enzymes. GDH and ALT activities were significantly elevated at protein levels of 44% and above, indicating enhanced hepatic capacity for deaminating excess amino acids and channeling their carbon skeletons into the citric acid cycle or gluconeogenesis [40]. This energetically costly shift aligns with the observed decreases in HSI and hepatic lipid at higher protein levels, suggesting the mobilization of lipid reserves to support amino acid processing. Concurrently, CPT‐1 activity increased significantly, peaking at 47%–50% protein. As the rate‐limiting enzyme for mitochondrial β‐oxidation, CPT‐1 upregulation suggests compensatory lipid catabolism to provide ATP for processing the high amino acid load [41]. This “protein‐induced lipid oxidation” creates an efficient metabolic state where amino acids are prioritized for growth, while lipids serve as primary fuel—a phenomenon validated in other animals [42]. Notably, lipogenic enzymes (FAS and ACC) were unaffected by dietary protein, confirming that the primary metabolic response to protein was catabolic and oxidative rather than lipogenic. This may be explained by increased branched‐chain amino acids promoting lipid oxidation through enhanced expression of lipid‐oxidizing enzymes [43]. In glucose metabolism, increased PEPCK and PFK‐1 activities with dietary protein implicate heightened gluconeogenic and glycolytic potential, likely utilizing amino acid‐derived carbon, as similarly observed in South American catfish *Rhamdia quelen* [44]. Thus, the enzyme profile at 44%–47% protein depicts a metabolically balanced liver that efficiently partitions nutrients: amino acids for growth, lipids for energy, and maintenance of glucose homeostasis.

In Experiment II, holding protein constant at 46% revealed how lipid level fine‐tunes metabolism. The most pronounced effect was on lipogenic enzymes: FAS and ACC exhibited significant downregulation with increasing dietary lipid—a classic feedback inhibition where ample dietary lipid suppresses hepatic de novo lipogenesis [45, 46]. In contrast, CPT‐1 activity showed a significant increasing trend, particularly at 13.5% lipid, suggesting that as lipid intake surpassed the optimal level for deposition, the liver upregulated fatty acid oxidation to prevent excessive accumulation. Activities of protein catabolic enzymes (GDH, ALT, and AST) were significantly suppressed in groups fed ≥10.5% lipid, consistent with the protein‐sparing effect discussed earlier. However, the fact that significant suppression occurred only above the optimal lipid level (10.5%) warrants attention. This may suggest that while adequate lipid spares protein, the further enzymatic suppression at higher levels reflects an altered metabolic state rather than an enhanced sparing effect—consistent with maintained high PRE at 9%–10.5% lipid and its subsequent decline at higher levels. Interestingly, PFK‐1 activity, a key glycolytic enzyme, exhibited a similar declining trend with increasing dietary lipid, with the lowest activities observed at lipid levels ≥10.5% (Table 6). This parallel suppression of both glycolytic and amino acid catabolic enzymes suggests a coordinated metabolic downregulation of glucose and amino acid utilization when abundant lipid is available as an alternative energy source, aligning with the classic glucose‐sparing and protein‐sparing actions of lipid [14, 47, 48].

This enzymatic profile confirms that the optimal protein and lipid levels establish a balanced metabolic state where amino acid catabolism is neither excessive nor insufficient, fatty acid oxidation is adequately primed, and lipogenesis is suppressed.

### 4.6. Hepatic Antioxidant Status

Evaluation of hepatic antioxidant status reveals that metabolic health is differentially modulated by dietary macronutrient composition, providing physiological context for interpreting growth and metabolic responses. In Experiment I, T‐AOC—an integrative biomarker reflecting combined enzymatic and nonenzymatic antioxidant capacity [49–51]—peaked at the 47% protein level, precisely aligning with the optimal level for growth. This suggests that this formulation simultaneously supports efficient anabolism and robust antioxidant defense. GSH‐Px activity remained elevated across the 41%–47% protein range, indicating enhanced capacity to detoxify peroxides, consistent with findings in tilapia and yellow catfish *Pelteobagrus fulvidraco* [52, 53]. This may involve activation of the Nrf2‐Keap1 signaling pathway, as demonstrated in hybrid grouper [50], offering a plausible molecular mechanism for the protein‐mediated enhancement. Notably, MDA levels remained stable across all protein groups, indicating that lipid peroxidation was effectively contained even at high‐protein intakes. The maintained oxidative balance at 47% protein underscores that this level is physiologically sustainable, meeting heightened metabolic demands without imposing a significant oxidative cost.

In contrast, Experiment II revealed a dose‐dependent pro‐oxidant effect of increasing dietary lipid. At the highest levels (12.5% and 14%), T‐AOC declined progressively, and SOD and GSH‐Px activities decreased significantly, indicating systemic suppression of the enzymatic antioxidant defense. This compromised state was directly linked to measurable oxidative damage as evidenced by elevated MDA in these groups. Crucially, hepatic UFA content increased at high lipid levels, suggesting that elevated MDA resulted not from UFA depletion but from enrichment of oxidizable substrates. The weakened antioxidant system was likely overwhelmed by increased ROS production accompanying intensified β‐oxidation [54]. This combination‐increased UFA substrates and compromised defenses—created an ideal environment for lipid peroxidation. The resulting MDA accumulation and peroxidative damage to cellular membranes likely contributed to the hepatocellular ballooning and vacuolization observed histologically (Section 4.2). The reduction in GSH‐Px is particularly significant as this enzyme neutralizes lipid hydroperoxides; its reduced activity impairs the cell’s ability to halt peroxidation chain propagation. This lipid‐induced oxidative stress, characterized by weakened antioxidant defenses and enhanced peroxidation potential, provides a mechanistic explanation for metabolic dysfunction at high lipid inclusions [45].Thus, while growth tolerates lipid up to 10.5%, antioxidant status indicates that levels at or below 10% are necessary to prevent oxidative stress, providing a health‐based criterion that complements the growth‐based recommendation.

### 4.7. Limitations and Perspectives

Several methodological limitations should be acknowledged. Protein and lipid were tested in separate univariate experiments rather than in a factorial design. While this stepwise approach is standard for establishing baseline requirements, it does not exclude the possibility that other protein–lipid combinations (e.g., 43% protein/13% lipid) might also support adequate growth. Thus, the optimal pair identified here (46% protein and 9.7%–10.4% lipid) represents a robust node under our test conditions but not necessarily the sole optimal formulation.

The use of microcrystalline cellulose as an inert filler to maintain isoenergetic conditions may have introduced a confounding effect as the best‐performing diets contained ~6% cellulose, whereas poorer‐performing diets contained substantially higher levels (approximately >20%). Given that cellulose is considered virtually indigestible for most carnivorous fish [55, 56], reduced dietary dilution may have partly contributed to the superior growth at intermediate nutrient levels. However, because cellulose inclusion was consistent across diets within each experiment and its levels in the optimal diets were low, the relative comparisons among treatments remain valid, and this factor does not undermine the practical relevance of our recommendations.

The absence of digestibility data precludes the calculation of digestible protein and digestible energy values. We therefore used crude protein and gross energy to derive the CP/GE ratio for practical formulation purposes, following the framework of Tibbetts et al. [57]. Future factorial studies combined with digestibility trials using practical cellulose‐free formulations would be valuable to confirm these optimal ranges under production‐relevant conditions and to establish digestible nutrient requirements. Despite these limitations, the present findings provide a solid baseline for practical feed development.

## 5. Conclusion

Given that the ultimate goal of fish nutrition research is to support the aquaculture industry in providing high‐quality protein for human consumption, this study establishes the recommended dietary protein and lipid requirements for juvenile greenfin filefish using practical feed formulations. Growth trials indicate that while a protein‐sparing effect is achievable with moderate lipid supplementation, excessive levels (>12%) trigger metabolic disorders, and high carbohydrate (>33%) disrupts metabolic homeostasis. These constraints collectively define the upper limits for nonprotein energy sources. Accordingly, a practical diet containing 46% protein, 9.7%–10.4% lipid, and ≤33% carbohydrate (corresponding to a CP/GE ratio of 23.93–24.59 mg/kJ) is recommended for greenfin filefish to ensure efficient biomass production, lean body composition, and metabolic health. These findings provide a nutritional framework for developing species‐specific feeds for the emerging aquaculture of greenfin filefish and may contribute to more sustainable and cost‐effective feed formulations. Future factorial studies may further explore alternative protein–lipid combinations that could achieve comparable performance.

## Author Contributions

**Zhidong Song**: experimental design and execution, writing – original draft. **Jian Zhang**: experimental design and execution, funding acquisition. **Peiyu Li**: feeding management, body composition determination, enzyme activity analysis. **Huadong Li, Lei Yu, Yuming Sun, and Yongzhi Sun**: diet preparation; **Xihan Xu, Yige Wu, Xiaoyan Wang, and Chengqiang Wang**: feeding management.

## Funding

This study was financially supported by the Natural Science Foundation of Shandong Province (Grant ZR2022MC132).

## Disclosure

All authors have read and agreed to the published version of the manuscript.

## Ethics Statement

All animal handling and experimental procedures were approved by the Animal Care and Use Committee of Shandong Marine Resource and Environment Research Institute and were conducted in accordance with the guidelines for the care and use of laboratory animals.

## Conflicts of Interest

The authors declare no conflicts of interest.

## Data Availability

The data that support the findings of this study are available from the corresponding author upon reasonable request.
